# The impact of SARS-CoV-2 on healthcare workers of a large University Hospital in the Veneto Region: risk of infection and clinical presentation in relation to different pandemic phases and some relevant determinants

**DOI:** 10.3389/fpubh.2023.1250911

**Published:** 2023-11-30

**Authors:** Filippo Liviero, Anna Volpin, Patrizia Furlan, Monica Battistella, Alessia Broggio, Laura Fabris, Francesco Favretto, Paola Mason, Silvia Cocchio, Claudia Cozzolino, Vincenzo Baldo, Angelo Moretto, Maria Luisa Scapellato

**Affiliations:** ^1^Department of Cardiac, Thoracic, and Vascular Sciences and Public Health, University of Padova, Padova, Italy; ^2^Occupational Medicine Unit, University Hospital of Padova, Padova, Italy; ^3^Preventive Medicine and Risk Assessment Unit, University Hospital of Padova, Padova, Italy

**Keywords:** COVID-19, healthcare personnel, health protection measures, asymptomatic infection, vaccines, contact tracing, biological risk, SARS-CoV-2 variants

## Abstract

**Aim:**

The aim of this study is to evaluate the incidence of SARS-CoV-2 infection and the prevalence of COVID-19-related symptoms in relation to pandemic phases and some relevant variables in a cohort of 8,029 HCWs from one of the largest Italian University Hospitals.

**Methods:**

A single-center retrospective study was performed on data collected during SARS-CoV-2 infection surveillance of HCWs. Cox’s multiple regression was performed to estimate hazard ratios of SARS-CoV-2 infection. Logistic multivariate regression was used to assess the risk of asymptomatic infections and the onset of the most frequent symptoms. All analyses were adjusted for sociodemographic and occupational factors, pandemic phases, vaccination status, and previous infections.

**Results:**

A total of 3,760 HCWs resulted positive (2.0%–18.6% across five study phases). The total incidence rate of SARS-CoV-2 infection was 7.31 cases per 10,000 person-days, significantly lower in phase 1 and higher in phases 4 and 5, compared to phase 3. Younger HCWs, healthcare personnel, and unvaccinated subjects showed a higher risk of infection. Overall, 24.5% were asymptomatic infections, with a higher probability for men, physicians, and HCWs tested for screening, fully vaccinated, and those with previous infection. The clinical presentation changed over the phases in relation to vaccination status and the emergence of new variants.

**Conclusion:**

The screening activities of HCWs allowed for the early detection of asymptomatic cases, limiting the epidemic clusters inside the hospital wards. SARS-CoV-2 vaccination reduced infections and symptomatic cases, demonstrating again its paramount value as a preventive tool for occupational and public health.

## Introduction

The outbreak of the novel coronavirus, “severe acute respiratory syndrome coronavirus 2” (SARS-CoV-2), began in Wuhan, Hubei Province, China, in December 2019. The resulting pandemic has caused significant morbidity and mortality, with over 764 million infections and over 6.9 million deaths reported globally as of 27 April 2023 (1). Italy was one of the first countries affected: since the start of the epidemic, over 25,9 million cases have been diagnosed and reported to the COVID-19 integrated surveillance system, with over 188,000 deaths (2). The global COVID-19 health emergency required unprecedented measures to control the spread of the virus, primarily through social distancing and mass quarantine, until vaccines against COVID-19 became available. Vaccination campaigns against SARS-CoV-2 began in several countries, including Italy, in December 2020. Priority was given to healthcare workers (HCWs) because of two main reasons. One reason relates to the fact that HCWs are at high risk of infection (3, 4), and infection among HCWs represents a matter of public health concern because they may have a role in spreading the disease among patients or colleagues, resulting in increased transmission in the community. In fact, in the early phase of the COVID-19 epidemic, several outbreaks of nosocomial transmission of SARS-CoV-2 infection have been documented involving patients, HCWs and other hospital staff, and subjects of the general population who came into close contact with hospital cases (5). Second, a significant transmission of infection among HCWs and their absence from work can also lead to a shortage of skilled personnel, given the increased demand for HCWs and hospital care during the pandemic (6). To date, over 479,835 cases have been diagnosed among Italian HCWs, with over 12,354 hospital admissions (7). For HCWs, the symptoms of SARS-CoV-2 infection, as well as those of the general population, were initially more severe and mainly involved the respiratory tract (5). The development of effective vaccines at the end of 2020 had a major impact on the clinical burden of COVID-19, reducing the cases of infection, preventing progression to serious and symptomatic forms of the disease, and reducing mortality (8–10). In Italy, during the COVID-19 pandemic, Law 76 of 28 May 2021 made vaccination mandatory for all HCWs. If they did not comply, they could be suspended from their profession (11, 12). Despite the positive impact of COVID-19 vaccines, the emergence of variants of concern with particular regard to Delta and Omicron since 2021 remains a challenge in controlling the spread of the virus and limits the efficacy of the vaccines (13). Some studies investigated the incidence of SARS-CoV-2 breakthrough infections (BIs) in HCWs and their determinants (14, 15). Earlier studies (13, 14, 16) found that previous SARS-CoV-2 infection and the standardized antibody titer were inversely related to the risk of BI. In particular, individuals with chronic diseases such as hypertension or cardiovascular diseases may have a lower serological response to vaccines administered for SARS-CoV-2 (17) and thus an increased risk of BI. Instead, the risk of BIs after a booster dose is significantly reduced by previous infection, heterologous vaccination, and older ages. Time elapsed from the booster affects BI severity, confirming the public health usefulness of the booster (18). To date, vaccines are still associated with a lower rate of hospitalization and milder forms of the disease, frequently leading to paucisymptomatic infection (14, 19). Since the beginning of the pandemic with vaccination, immunity from previous infection, and the evolution of new variants that cause less intense acute infection, the presentation of symptoms has evolved (20). To the best of our knowledge, there are no studies that have investigated the trend of the SARS-CoV-2 infection and clinical presentation in HCWs for a long period covering different pandemic phases (i.e., from 17 February 2020 to 06 June 2022), in relation to some relevant determinants. The analysis of the trend of SARS-CoV-2 infections in HCWs over a long time across different pandemic phases could help to understand better and evaluate the role of some infection prevention and control measures such as hospital screening activities, contact tracing, and vaccination, which could be useful to implement, for example, in future epidemic exacerbations, to reduce the spread of contagion in working and living environments. Thus, this study aimed to evaluate the incidence of SARS-CoV-2 infection in a cohort of HCWs from a large University Hospital in the Veneto Region, northeastern Italy, and the probability of occurrence of asymptomatic infections among positive HCWs in relation to some demographic and occupational characteristics, different pandemic phases, vaccination status, and previous infections. The prevalence of different COVID-19-related symptoms was also investigated in relation to the aforementioned variables.

## Materials and methods

### Study design

A single-center retrospective observational study was performed on data collected during the risk management of SARS-CoV-2 infection and surveillance of HCWs from Azienda Ospedale-Università Padova (AOUP).

### Setting

AOUP is one of the largest University Hospitals in Italy, with 1,700 beds, 70,000 recovery, and 7 million outpatient specialist procedures performed every year in close collaboration with the University of Padova. AOUP employs more than 8,000 operators, including physicians, residents, nurses, allied health professionals, and technical and administrative staff, who assist in more than 100 different units. During COVID-19, AOUP was identified as a regional emergency hub. In early February 2020, the Hospital Direction of AOUP activated a crisis unit and, based on the rapid evolution of the epidemiological scenario, undertook a major reorganization to increase the wards’ capacity to admit COVID-19 patients and the availability of dedicated healthcare staff. A detailed description of the organizational and management measures implemented by AOUP in relation to the COVID-19 pandemic has been previously reported (21).

### Study period, sample and data collection, and inclusion/exclusion criteria

The information systematically collected during the HCWs’ surveillance of SARS-CoV-2 infection during the period 24 February 2020–06 June 2022 was retrospectively analyzed. AOUP personnel have been subjected to periodic screening tests for SARS-CoV-2 since 18 March 2020, with timing determined by the hospital management based on the epidemiological trend of the pandemic and the recommendations from the Regional Directorate of Prevention, Food Safety—Public Health of the Veneto Region. SARS-CoV-2 infections were diagnosed by positive real-time reverse-transcriptase polymerase chain reaction (rt-PCR) on nasopharyngeal swabs and from August 2021 on saliva samples alternatively described elsewhere (22, 23). Based on the epidemiological trend of the infections, the introduction of vaccination, and the emergence of the variant of concerns, five study phases have been identified as follows: the first between 17 February 2020 and 19 July 2020, the second between 20 July 2020 and 31 January 2021, the third between 01 February 2021 and 31 October 2021, the fourth between 01 November 2021 and 28 February 2022, and the fifth between 01 March 2022 and 06 June 2022. The beginning of the second phase was identified with the resumption of cases after a period of absence of infections among HCWs (the last case of the first phase was on 10 May 2020); the beginning of the third phase was identified with the start of the administration of the second dose of vaccine for SARS-CoV-2 (the vaccination campaign started in AOUP on 27 December 2020 with the administration of the Comirnaty Pfizer m-RNA vaccine-Biontech); the fourth phase was defined in relation to the predominance of the Delta variant and the administration of the booster dose of the vaccine (third dose); and the fifth was associated with the spread of the Omicron variant.

HCWs were included in the study if routinely tested at the phases in which they were present, while those absent for the entire period and those not yet vaccinated (at least one dose) at the end of the study period were excluded from the analysis. Unvaccinated HCWs, according to Italian legislation (Law 76 of 28 May 2021), could not have a job position that presented a risk of spreading the infection.

During the SARS-CoV-2 infection surveillance of HCWs, a 24-h telephone triage was carried out to provide information support to HCWs, trace close contacts of suspected or confirmed COVID-19 cases according to international, national, and local guidelines (24), and collect some other information on symptoms and vaccination status. Additional sociodemographic information was retrieved from hospital databases made available to occupational physicians who carry out the health surveillance activities of HCWs according to current Italian legislation (Legislative Decree 81/08). The following information was obtained from these databases and by contact tracing activity: sex, age, job-title, working in a COVID area, presence of clusters, swab motivation (by contact with an infected patient or colleague, outwork contact, and test performed for screening or any symptom), vaccination status, and pandemic phase. We categorized the job titles as physicians, residents, nurses, allied health professionals, other healthcare personnel (e.g., radiology technicians and laboratory technicians), and other non-healthcare personnel (e.g., administrative staff and others).

For SARS-CoV-2 positive HCWs, information on symptomatic infection (yes/no), type of symptoms, and previous infections were also collected. HCWs without symptoms at the time of the positive swab and who continued to remain symptom-free during the isolation period were considered asymptomatic. During contact tracing activity, the following symptoms were referred by positive HCWs: fever, sore throat, cough, dyspnea, rhinorrhea and nasal obstruction, headache, ageusia/anosmia, asthenia, myalgia/arthralgia, nausea/vomiting, diarrhea, anorexia, chest pain, and mental confusion.

We categorized the timing of previous infection as “no previous infection,” “≤12 months,” and “12+ months.”

We considered as vaccinated those individuals who received the first vaccine dose 14 or more days before infection.

Data were anonymized and entered into an *ad hoc* database. The research was performed following the 1964 Declaration of Helsinki standards and its later amendments and was approved by both the Ethics Committee of the Italian National Institute of Infectious Diseases (INMI) Lazzaro Spallanzani and the local Ethics Committee (288n/AO/22).

### Statistical analysis

A descriptive analysis was conducted on HCWs’ demographic, occupational, and clinical data. Data were presented as percentages for categorical variables or as means ± standard deviation (SD) for continuous variables. The continuous variables were compared using Student’s t-test for unpaired data, performing *a priori* test for equality of variances. To evaluate the incidence of SARS-CoV-2 infection among HCWs, a Cox’s multiple regression model was performed. For each subject, their follow-up was computed as the number of days that elapsed between the entry date (starting date of the study period or the work) and the exit date (date of infection or ending date of the study period or drop-out from follow-up, whichever came first). The incidence rate was calculated by dividing the number of positive tests by the total person-time expressed per 10,000 person-days. Thus, Cox’s regression analysis was used to estimate hazard ratios (HR) of SARS-CoV-2 infection in the study period, considering the presence of an infection as the dependent event and adjusting for potential confounding factors such as sex, age, occupational characteristics (job title, working in a COVID area, and presence of clusters), pandemic phases, and vaccination status. The adjusted HRs (adj) and 95% confidence intervals (95%CI) were estimated. To assess the risk of asymptomatic/symptomatic infections among positive HCWs and to assess the risk of the onset of the most frequent symptoms, a logistic multivariate regression was performed. The following covariates were considered: sex, age, job title, working in a COVID area, presence of clusters, swab motivation, vaccination status, previous SARS-CoV-2 infection, and pandemic phase. Regarding symptoms, the covariates included in the model were sex, age, vaccination status, previous infection, and pandemic phase. The adjusted ORs (adj) and 95% confidence intervals (95%CI) were estimated. A value of p of < 0.05 was accepted as statistically significant. Statistical analyses were performed using SPSS Statistics, version 28.0.

## Results

**Figure 1:**
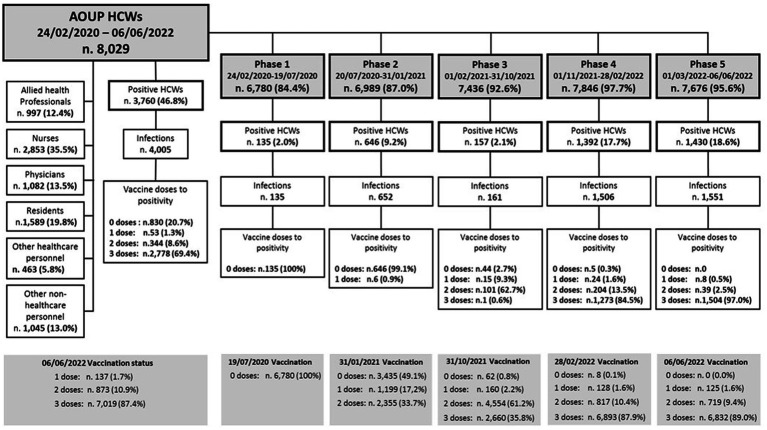
Distribution of AOUP HCWs by job title, pandemic study phases, vaccination status, and SARS-CoV-2 infections.

A total of 8,029 HCWs permanently employed in AOUP and routinely tested for SARS-CoV-2 in the study period were included in the analysis. The distribution of HCWs by job title, study phases, prevalence of SARS-CoV-2 infections, and vaccination status to positivity are shown in Figure 1. Physicians, residents, and nurses represented more than two-thirds of HCWs tested for SARS-CoV-2. The remaining personnel included allied health professionals (12.4%) and employed in other jobs (18.8%), identified as “other healthcare personnel” and “other non-healthcare personnel” (i.e., administrative staff and technical workers). Key characteristics of the study population are included in Table 1. More than three-quarters of the study population were over 30 years old, and women represented more than two-thirds of HCWs included in the analysis (which reflects the HCWs demographics in Italy). Overall, the mean age was 43.7 ± 12.3 years, with a slightly but significantly lower mean age in men (42.7 ± 13.1 vs. 44.2 ± 11.9 years; *p* < 0.001). At the end of the study period, most HCWs were vaccinated with one dose (1.7%), two doses (10.9%), and three doses (87.4%). In particular, during phase 1, no HCWs were vaccinated as no vaccine was available. During phase 2, 50.9% of HCWs were vaccinated (17.2% with one dose and 33.7% with two doses), and nearly all (99.2%) by the end of phase 3 (61.2% with two doses and 35.8% with three doses). At the end of phases 4 and 5, 87.9 and 89.0% of HCWs were vaccinated with three doses, respectively (Figure 1). Overall, 3,760 HCWs (i.e., 46.8% of HCWs tested) resulted positive in a total of 4,005 infections in the study period. The number of HCWs with multiple infections was 240, for a total of 485 infections. Of these, 236 had a second positivity with a mean elapsed time of 372 days, while three subjects tested positive three times and one subject four times. In phase 1, positive HCWs accounted for 2.0%, in phase 2 for 9.2%, in phase 3 for 2.1%, in phases 4 and 5 for 17.7 and 18.6%, respectively (Figure 1; Table 1). At the time of their first positivity, 20.7% of HCWs were not vaccinated, 1.3% had received a single dose of vaccine, 8.6% had two doses of vaccine, and 69.4% also had the booster dose (Figure 1). Overall, there was an incidence rate of SARS-CoV-2 infections of 7.31 cases per 10,000 person-days, without significant sex differences. In particular, the lowest incidence rate was recorded in phase 3 and among workers with two doses of vaccine, while the highest incidence rate was recorded in phases 4 and 5 (Table 1). Multivariate analysis showed a significant risk of infection for younger workers and healthcare personnel (i.e., allied health professionals, nurses, physicians, and residents). With regard to the pandemic phase, the risk of infection increased over the study period, resulting in significantly lower in phase 1 and higher in phases 4 and 5, compared to phase 3. Interestingly, the risk of infection for HCWs with two doses of vaccine was significantly lower compared to unvaccinated workers. In addition, HCWs who had received the booster dose also showed a significantly lower risk of infection compared to the unvaccinated HCWs. However, this reduction in the risk of infection was lower in HCWs with booster doses compared to HCWs with two doses of vaccine.

**Table 1:** Incidence rates of SARS-CoV-2 infections among HCWs according to selected characteristics.

	HCWs (n.8,029)		Positive HCWs (n.3,760)		Follow-up (days-person)	Incidence × 10,000	adjHR (IC95%)
	*N*	(%)	*N*	(%)			
**Gender**							
*M*	2,512	(31.3)	1,175	(31.3)	1,561,179	7.53	0.99 (0.92–1.06)
*F*	5,517	(68.7)	2,585	(68.8)	3,583,499	7.21	(ref)
**Age groups**							
*≤30*	1,896	(23.6)	1,143	(30.4)	931,189	12.27	**2.34 (2.10–2.60)**
*31–49*	2,914	(36.3)	1,415	(37.6)	1,879,032	7.53	**1.51 (1.39–1.63)**
*50+*	3,219	(40.1)	1,202	(32.0)	2,334,457	5.15	(ref)
**Job title**							
*Allied health professionals*	997	(12.4)	495	(13.2)	647,331	7.65	**1.62 (1.41–1.85)**
*Nurses*	2,853	(35.5)	1,359	(36.1)	1,937,190	7.02	**1.28 (1.14–1.44)**
*Physicians*	1,082	(13.5)	489	(13.0)	706,054	6.93	**1.35 (1.18–1.55)**
*Residents*	1,589	(19.8)	868	(23,1)	801,632	10.83	**1.23 (1.06–1.41)**
*Other healthcare personnel*	463	(5.8)	179	(4.8)	319,697	5.60	1.01 (0.84–1.21)
*Other non-healthcare personnel*	1,045	(13.0)	370	(9.8)	732,774	5.05	(ref)
**Pandemic phase**							
*Phase 1*	6,780	(84.4)	135	(3.6)	968,365	1.39	**0.35 (0.26–0.49)**
*Phase 2*	6,989	(87.0)	646	(17.2)	1,279,045	5.05	1.11 (0.84–1.487)
*Phase 3*	7,436	(92.6)	157	(4.2)	1,727,458	0.91	(ref)
*Phase 4*	7,846	(97.7)	1,392	(37.0)	734,465	18.95	**16.79 (13.32–21.17)**
*Phase 5*	7,676	(95.6)	1,430	(38.0)	435,334	32.85	**29.84 (23.55–37.80)**
**Vaccination status (n. of doses)**							
0	7,090	(88.3)	821	(21.8)	2,282,299	3.60	(ref)
1	6,286	(78.3)	36	(1.0)	155,919	2.31	**0.38 (0.26–0.57)**
2	6,723	(83.7)	301	(8.0)	1,720,784	1.75	**0.14 (0.11–0.19)**
3	6,174	(76.9)	2,602	(69.2)	985,665	26.40	**0.36 (0.26–0.49)**

Cox’s multiple regression model. Bold indicates statistically significant results. adjHR, adjusted Hazard Ratio; (IC95%), 95% Confidence Interval; ref, reference.

In Table 2, the totality of SARS-CoV-2 infections is stratified for the different characteristics of interest. More than three-quarters of the 4,005 infections presented at least one symptom attributable to COVID-19, while 24.5% were asymptomatic infections. Multivariate analysis showed a significant increase in the probability of being asymptomatic for men, physicians, HCWs tested for screening, primary-cycle vaccinated HCWs and booster dose recipients, and those HCWs who have already had previous infection (≤12 months and >12 months). With regard to the pandemic phase, a higher probability of being asymptomatic was found during phase 1, compared to phase 5. However, phases 2, 3, and 4 showed a significant increase in the probability of being asymptomatic compared to phase 5, although lower than that is seen in phase 1.

**Table 2:** Distribution of HCWs testing positive for SARS-CoV-2 and multivariate logistic regression analysis investigating some relevant characteristics and symptoms.

	HCWs (n. 3,760)		Positivity to SARS-CoV-2	Asymptomatic HCWs (n.981)		adjOR (IC95%)
	n.	(%)	(n. 4,005)	n.	(%)	
**Gender**						
*Male*	1,175	(31.3)	1,236	369	(29.9)	**1.39 (1.17–1.65)**
*Female*	2,585	(68.8)	2,769	612	(22.1)	(ref)
**Age groups**						
*≤30*	1,143	(30.4)	1,203	290	(24.1)	0.80 (0.62–1.04)
*31–49*	1,414	(37.6)	1,527	382	(25.0)	1.01 (0.83–1.22)
*50+*	1,203	(32.0)	1,275	309	(24.2)	(ref)
**Job title**						
*Allied health professionals*	495	(13.2)	548	122	(22.3)	(ref)
*Nurses*	1,359	(36.1)	1,460	317	(21.7)	1.17 (0.91–1.52)
*Physicians*	489	(13.0)	514	159	(30.9)	**1.71 (1.26–2.33)**
*Residents*	868	(23.1)	912	241	(26.4)	**1.61 (1.15–2.243)**
*Other healthcare personnel*	179	(4.8)	189	46	(24.3)	1.30 (0.85–2.00)
*Other non-healthcare personnel*	370	(9.8)	382	96	(25.1)	1.33 (0.95–1.86)
**Swab motivation**						
*Contact with positive HCW*	314	(8.4)	324	94	(29.0)	**1.37 (1.01–1.86)**
*Contact with positive patient*	183	(4.9)	196	50	(25.5)	1.27 (0.87–1.84)
*Outwork contact*	1,327	(35.3)	1,417	308	(21.7)	(ref)
*Screening*	1,079	(28.7)	1,167	493	(42.2)	**2.90 (2.42–3.48)**
Non screening^*^	857	(22.8)	901	36	(4.0)	**0.17 (0.12–0.24)**
**Cluster**						
*Yes*	551	(14.7)	574	139	(24.2)	**0.70 (0.55–0.89)**
*NO*	3,209	(85.3)	3,431	842	(24.5)	(ref)
**COVID area**						
*Yes*	553	(14.7)	589	151	(25.6)	0.94 (0.75–1.17)
*NO*	3,207	(85.3)	3,416	830	(24.3)	(ref)
**Vaccination status to positivity (N. of doses)**						
*0*	821	(21.8)	830	245	(29.5)	(ref)
*1*	36	(1.0)	53	15	(28.3)	1.69 (0.65–4.40)
*2*	301	(8.0)	344	99	(28.8)	**2.56 (1.10–5.93)**
*3*	2,602	(69.2)	2,778	622	(22.4)	**2.49 (1.04–5.99)**
**Previous infections**						
None	3,520	(93.6)	3,760	905	(24.1)	(ref)
*≤12 months*	82	(2.2)	86	29	(33.7)	**1.80 (1.09–2.98)**
*12+ months*	158	(4.2)	159	47	(29.6)	**1.51 (1.02–2.23)**
**Pandemic study phase**						
*Phase1*	135	(3.6)	135	65	(48.1)	**14.03 (5.35–36.84)**
*Phase2*	646	(17.2)	652	176	(27.0)	**6.03 (2.45–14.82)**
*Phase3*	157	(4.2)	161	41	(25.5)	**2.61 (1.53–4.44)**
*Phase4*	1,392	(37.0)	1,506	456	(30.3)	**2.20 (1.82–2.67)**
*Phase5*	1,430	(38.0)	1,551	243	(15.7)	(ref)

^*^Test performed for symptoms; bold indicates statistically significant results. adjOR, adjusted Odds Ratio; (IC95%), 95% Confidence Interval; ref, reference.

Table 3 shows the distribution of HCWs’ self-reported source of contact and exposure circumstances. Approximately one-third (35.4%) of the total SARS-CoV-2 infections occurred outside of the workplace, while 13% were by contact with a positive patient or a positive colleague (4.9% and 8.1%, respectively). The source of contact was unknown for 51.6% of infections and emerged during the hospital screening activity (29.1%) or tests executed in symptomatic HCWs (22.5%). Interestingly, the percentage of HCWs infected outside of the workplace increased from 15.6% in phase 1 up to 48.4% in the next phases, while the percentage of HCWs infected in the workplace (by contact with a positive colleague or with a positive patient) was higher in phase 1 and progressively decreased in the next phases (Table 3).

**Table 3:** Distribution of HCWs self-reported source of contact and swab motivation.

	Phase 1		Phase 2		Phase 3		Phase 4		Phase 5		Total	
	*N*	%	*N*	%	*N*	%	*N*	%	*N*	%	*N*	%
*Outwork contact*	21	15.6	208	31.9	78	48.4	602	40.0	508	32.8	1,417	35.4
*Contact with positive patient*	15	11.1	83	12.7	8	5.0	52	3.5	38	2.5	196	4.9
*Contact with positive HCW*	49	36.3	72	11.0	8	5.0	107	7.1	88	5.7	324	8.1
*Screening*	32	23.7	151	23.2	32	19.9	510	33.9	442	28.5	1,167	29.1
Non-screening^*^	18	13.3	138	21.2	35	21.7	235	15.6	475	30.6	901	22.5
Total	135	100.0	652	100.0	161	100.0	1,506	100.0	1,551	100.0	4,005	100.0

^*^Test performed for symptoms.

Multivariate analysis (see Supplementary Table S1) showed a significant increase in the risk of infection from contact with positive patients in workers in the ≤30 years age class. HCWs in the 31–49 years and +50 years age classes were more likely to be infected out of the workplace, instead. Regarding the job title, allied health professionals had a significantly higher probability of infection by contact with positive patients, while nurses were from contacts out of the workplace. Residents and other non-healthcare personnel showed a significant increase in the infection risk by contact with a positive colleague. In addition, the probability of infection by contact with a positive patient or colleague was significantly higher within a cluster. Finally, the probability of infection by contact with a positive colleague was also significantly higher in the non-COVID area and during phase 1 (Supplementary Table S1).

The most frequent symptoms reported by HCWs during the acute phase of infection were fever (37.2%), sore throat (37.4%), cough (33.7%), and rhinorrhea (33.7%), followed by headache (19.0%), myalgia/arthralgia (16.0%), and nasal obstruction (14.7%; see Supplementary Table S2), some with significant variations between pandemic study phases. Multivariate analysis showed (Table 4) a significant risk of presenting ageusia/anosmia in all study phases, compared to phase 5, with a decreasing trend from phase 1 to phase 5. The probability of presenting fever was significantly higher in phase 5 and in non-vaccinated HCWs, while rhinorrhea/nasal obstruction in phases 2 and 3 and sore throat in phases 4 and 5 and among younger HCWs. The risk of presenting headache and myalgia/arthralgia was significantly higher in women, and changed among phases; in addition, the risk of myalgia/arthralgia was higher in the 50+ years age class.

**Table 4:** Distribution of COVID-19 symptoms and multivariate logistic regression analysis investigating some relevant characteristics.

	Symptomatic HCWs	Fever (n.1,125)			Cough (n.1,020)			Sore throat (n.1,132)			Anosmia/ageusia (n.152)		
	(n.3,024)	*N*	%	adjOR (95%CI)	*N*	%	adjOR (95%CI)	*N*	%	OR adjOR (95%CI)	*N*	%	adjOR (95%CI)
**Gender**													
Male	867	321	(37.0)	0.96 (0.81–1.13)	273	(31.5)	ref	320	(36.9)	ref	48	(5.5)	1.17 (0.80–1.71)
Female	2,157	804	(37.3)	ref	747	(34.6)	1.17 (0.99–1.39)	812	(37.6)	1.07 (0.90–1.26)	104	(4.8)	ref
**Age groups**													
≤30	913	338	(37.0)	1.15 (0.95–1.40)	322	(35.3)	1.12 (0.92–1.36)	379	(41.5)	**1.22 (1.01–1.48)**	42	(4.6)	ref
31–49	1,145	434	(37.9)	1.13 (0.94–1.36)	377	(32.9)	0.99 (0.83–1.19)	418	(36.5)	1.03 (0.86–1.24)	60	(5.2)	0.95 (0.61–1.47)
50+	966	353	(36.5)	ref	321	(33.2)	ref	335	(34.7)	ref	50	(5.2)	0.77 (0.49–1.22)
**N. of doses to positivity**													
0	585	331	(56.6)	**3.19 (1.45–7.02)**	189	(32.3)	0.94 (0.40–2.18)	94	(16.1)	0.60 (0.23–1.61)	115	(19.7)	**6.49 (1.54–27.31)**
1	38	10	(26.3)	0.87 (0.40–1.86)	13	(34.2)	1.16 (0.57–2.37)	17	(44.7)	1.49 (0.74–3.01)	1	(2.6)	2.17 (0.26–18.28)
2	245	87	(35.5)	1.32 (0.94–1.84)	83	(33.9)	1.15 (0.83–1.60)	68	(27.8)	**0.67 (0.48–0.93)**	19	(7.8)	**6.50 (2.88–14.72)**
3	2,156	697	(32.3)	ref	735	(34.1)	ref	953	(44.2)	ref	17	(0.8)	ref
**Previous infections**													
None	2,855	1,077	(37.7)	ref	958	(33.6)	ref	1,056	(37.0)	ref	149	(5.2)	ref
≤12 months	57	18	(31.6)	0.79 (0.44–1.41)	20	(35.1)	1.01 (0.58–1.77)	22	(38.6)	0.96 (0.55–1.69)	2	(3.5)	0.81 (0.18–3.64)
1 + months	112	30	(26.8)	0.74 (0.48–1.14)	42	(37.5)	1.14 (0.77–1.68)	54	(48.2)	1.23 (0.84–1.80)	1	(0.9)	0.63 (0.08–4.76)
**Pandemic study phase**													
Phase 1	70	52	(74.3)	2.41 (0.94–6.21)	28	(40.0)	1.58 (0.59–4.14)	9	(12.9)	0.88 (0.29–2.65)	17	(24.3)	**10.19 (1.92–54.25)**
Phase 2	476	255	(53.6)	0.96 (0.43–2.12)	151	(31.7)	1.09 (0.46–2.58)	79	(16.6)	1.18 (0.49–2.85)	94	(19.7)	**7.81 (1.59–38.42)**
Phase 3	120	54	(45.0)	1.31 (0.78–2.20)	34	(28.3)	0.80 (0.47–1.37)	20	(16.7)	ref	12	(10.0)	**3.78 (1.08–13.27)**
Phase 4	1,050	297	(28.3)	ref	338	(32.2)	ref	424	(40.4)	**2.49 (1.34–4.61)**	22	(2.1)	**2.52 (1.01–6.27)**
Phase 5	1,308	467	(35.7)	**1.48 (1.23–1.77)**	469	(35.9)	**1.21 (1.01–1.44)**	600	(45.9)	**3.0 (1.59–5.64)**	7	(0.5)	ref

	Symptomatic HCWs	Rhinorrhea/nasal obstruction (n.1,406)			Headache (n.576)			Myalgia/arthralgia (n.483)			Asthenia (n.395)		
	(n.3,024)	*N*	%	adjOR (95%CI)	*N*	%	adjOR (95%CI)	*N*	%	adjOR (95%CI)	*N*	%	adjOR (95%CI)
**Gender**													
Male	867	392	(45.2)	ref	132	(15.2)	ref	115	(13.3)	ref	116	(13.4)	1.04 (0.82–1.32)
Female	2,157	1,014	(47.0)	1.10 (0.93–1.29)	444	(20.6)	**1.42 (1.15–1.76)**	368	(17.1)	**1.30 (1.03–1.64)**	279	(12.9)	ref
**Age groups**													
≤30	913	443	(48.5)	1.16 (0.96–1.39)	151	(16.5)	ref	110	(12.0)	ref	111	(12.2)	ref
31–49	1,145	541	(47.2)	1.13 (0.94–1.34)	225	(19.7)	1.17 (0.93–1.47)	184	(16.1)	1.28 (0.98–1.66)	142	(12.4)	1.00 (0.77–1.31)
50+	966	422	(43.7)	ref	200	(20.7)	1.21 (0.96–1.54)	189	(19.6)	**1.54 (1.19–2.01)**	142	(14.7)	1.19 (0.91–1.56)
**N. of doses to positivity**													
0	585	176	(30.1)	**0.24 (0.11–0.54)**	148	(25.3)	0.88 (0.37–2.11)	179	(30.6)	1.38 (0.57–3.35)	114	(19.5)	1.60 (0.55–4.68)
1	38	17	(44.7)	0.63 (0.31–1.25)	7	(18.4)	0.79 (0.32–1.94)	7	(18.4)	1.08 (0.44–2.67)	1	(2.6)	0.21 (0.03–1.56)
2	245	126	(51.4)	0.87 (0.63–1.19)	57	(23.3)	1.01 (0.71–1.56)	42	(17.1)	ref	27	(11.0)	0.93 (0.55–1.55)
3	2,156	1,087	(50.4)	ref	364	(16.9)	ref	255	(11.8)	**0.63 (0.41–0.96)**	253	(11.7)	ref
**Previous infections**													
None	2,855	1,321	(46.3)	ref	552	(19.3)	ref	459	(16.1)	ref	381	(13.3)	ref
≤12 months	57	26	(45.6)	0.94 (0.55–1.62)	10	(17.5)	0.96 (0.47–1.93)	5	(8.8)	0.48 (0.18–1.22)	5	(8.8)	0.73 (0.29–1.87)
12+ months	112	59	(52.7)	1.14 (0.77–1.67)	14	(12.5)	0.65 (0.37–1.16)	19	(17.0)	1.34 (0.80–2.25)	9	(8.0)	0.68 (0.34–1.36)
**Pandemic study phase**													
Phase 1	70	9	(12.9)	ref	14	(20.0)	1.50 (0.52–4.35)	9	(12.9)	ref	14	(20.0)	1.21 (0.36–4.05)
Phase 2	476	154	(32.4)	**3.21 (1.55–6.64)**	123	(25.8)	2.09 (0.84–5.18)	161	(33.8)	**3.55 (1.71–7.35)**	92	(19.3)	1.18 (1.40–3.46)
Phase 3	120	68	(56.7)	**3.73 (1.43–9.70)**	37	(30.8)	**2.43 (1.33–4.44)**	23	(19.2)	2.02 (0.73–5.55)	17	(14.2)	1.26 (0.57–2.77)
Phase 4	1,050	498	(47.4)	1.47 (0.50–4.28)	199	(19.0)	**1.26 (1.01–1.57)**	127	(12.1)	1.93 (0.60–6.19)	113	(10.8)	ref
Phase 5	1,308	677	(51.8)	1.71 (0.58–5.04)	203	(15.5)	ref	163	(12.5)	2.16 (0.66–7.04)	159	(12.2)	1.13 (0.87–1.47)

Bold indicates statistically significant results. adjOR, adjusted Odds Ratio; (IC95%), 95% Confidence Interval; ref, reference.

Overall, nine HCWs were hospitalized in phase 1 and six in phase 2, while no hospitalizations occurred in the other phases. Among the hospitalized subjects in phase 1, seven were admitted to a non-critical area for a limited period of time, one required semi-intensive therapy, and the last, affected by comorbidities (65-year-old men, suffering from hypertension and type II diabetes), developed severe acute respiratory failure (ARDS) and was admitted to the intensive care unit. Among the hospitalized HCWs of phase 2, five were hospitalized in non-critical areas and a 40-year-old worker, with no significant comorbidities, required a semi-intensive therapy for interstitial pneumonia, which evolved into ARDS. No deaths were reported in AOUP HCWs.

## Discussion

This study analyzed the risk of SARS-CoV-2 infection and the probability of having an asymptomatic infection among HCWs belonging to one of the largest Italian University Hospital (25) for a long pandemic period stretching up to 27 months, in relation to different pandemic phases (from 17 February 2020 to 06 June 2022) and some relevant determinants.

The main results of this analysis revealed that the prevalence of infection in AOUP HCWs varied across study phases, ranging from 2.0% to 18.6%. The incidence of SARS-CoV-2 infection was significantly lower in phase 1 and higher in phases 4 and 5, compared to phase 3. Younger HCWs (≤30 year age class), healthcare personnel, and unvaccinated subjects showed a higher risk of infection. Approximately a quarter of positive HCWs presented an asymptomatic infection that was influenced in this study population by the following determinants: being of male gender, physician, personnel tested for screening, primary-cycle vaccinated HCWs, booster dose recipients, and subjects with previous infection. A higher probability of being asymptomatic was found in phase 1, compared to phase 5. The clinical presentation of positivity changed over the study phases in relation to vaccination status and the emergence of new variants.

Overall, 35.4% of the total SARS-CoV-2 infections occurred outside of the workplace, while only a small part occurred in the workplace (i.e., 13%% by contact with a positive patient or by contact with a positive colleague), and more than half of the infections had an unknown source of contact. We can speculate that a considerable proportion of these cases with unknown sources of contact probably occurred outside of the workplace. Interestingly, during phase 1, the source of contact resulted unknown for 37% of infections. These cases emerged, for the most part, during the hospital screening activity that was promptly implemented for all AOUP HCWs on 18 March 2020. In fact, AOUP already had an emergency plan in place in early February 2020 and was able to activate the crisis unit as soon as the first positive case of COVID-19 was confirmed on 21 February 2020. To adapt to COVID-19’s rapid spread, the hospital has been reorganized to meet the key objectives, as described previously (21). During the early stages of phase 1, in relation to the occurrence of some SARS-CoV-2 clusters within some operating units and the analysis performed to reconstruct the transmission chains of the infections (see Supplementary Figure S1), strong evidence emerged that asymptomatic and pre-symptomatic subjects represented a significant risk for transmission. This was also observed by others both in the healthcare setting (26–28) and in the general population (22, 29). This observation prompted the application of infection control policies to protect HCWs and patients. In addition to the availability of protective devices and the implementation of safety protocols, the major challenge in preventing the spread of nosocomial is the prompt detection and isolation of asymptomatic individuals by screening campaigns. Despite the control measures taken during phase 1, AOUP HCWs showed to be more infected in the workplace (47.4% of the total number of contagions in this phase), due to the aforementioned clusters occurring in AOUP, and only 15.6% out of the workplace, probably in relation to the introduction of the lockdown measures in our country (in the period 09 March 2020–03 May 2020). However, in this phase, the prevalence of infection in AOUP (i.e., 2%) was lower than those reported in other Italian hospitals (30, 31). It should be noted that the lack of personal protective equipment (PPE) suffered in the early stages of the pandemic never occurred in AOUP, which always guaranteed them, at least in risky activities. Thus, the prevalence in AOUP was also the lowest among the hospitals of the Veneto Region (in which the mean prevalence was 5.5%) (32). At the University Hospital of Verona, where periodic screening of all HCWs was performed as per AOUP, the prevalence of infection was 4% (33). Lahner et al. (34) recorded a prevalence of 2.7% among all employees tested at University Hospital in Lazio, a region that was less affected than Veneto in the early stages of the pandemic (35). Moreover, an infection prevalence of 4.8% was reported at Cambridge University Hospital (36), 11.9% at a University Hospital in Madrid (37), and 9.0% at a hospital in Cleveland, Ohio (38). In addition, infections that occurred by confirmed contact with a positive patient in phase 1 (11.1%) were lower than those recorded during the same period in the hospitals of Turin (47.8%) (39), Milan (50%) (40), and Trieste (51.3%) (41). Overall, these data confirm the efficacy of the measures introduced in AOUP to limit the nosocomial spread of SARS-CoV-2 among HCWs. The next phases were characterized by a progressive decrease in viral transmission in the workplace and an increase in infections occurring outside of the workplace. It should be kept in mind that HCWs were exposed to the virus outside the workplace since the lockdown was no longer declared. In fact, phase 2 was signed by the rapid resumption of cases after a period of absence of infections among HCWs in a pre-vaccination era. In AOUP, the vaccination campaign started on 27 December 2020 (with the administration of the Comirnaty Pfizer m-RNA vaccine–BioNTech) and continued with the administration of the second dose at the end of phase 2 and in phase 3. In compliance with legislative decree 81/08, occupational physicians participated in this campaign, vaccinating HCWs (11). Italy, with Law 76/21, decided to make this vaccination mandatory for HCWs, following a different approach compared to many other European countries (12). In our study population, the lowest incidence rate of SARS-CoV-2 infections was recorded during phase 3 and among workers who received two doses of the vaccine. Indeed, vaccination reduced the transmission rates of SARS-CoV-2, particularly in the first 4–6 months after the vaccination, due to a more rapid decline in viral load and decreased viability of the virus shed by vaccinated individuals; indeed, it was less likely to raise a virus culture from swabs of these subjects (19). AOUP HCWs who had received the booster dose showed a less impressive reduction of the risk of infection compared to those with two doses of vaccine, although still significant compared with unvaccinated HCWs. Several reasons may be taken into account for this result. In fact, the continued emergence of new viral variants with different traits has both extended viral transmission and threatened the effectiveness of vaccines, boosting the risk of BI (13). Thus, the risk of testing positive was significantly higher in phases 4 and 5 compared to phase 3 due to the spread of the highly contagious variants (the Delta and the Omicron variants, respectively), despite the administration of the booster dose of the vaccine during phase 4. However, taking the period covering phases 1, 2, and 3, the prevalence of SARS-CoV-2 infections in AOUP was lower than those reported in the same period at the Trieste University Hospital, North East of Italy (42), and in line with those reported at the University Health Agency Giuliano-Isontina (ASUGI) that, however, analyzed data from 1 March 2020 to 31 May 2022 (43). Furthermore, during phases 2 and 3, the infection prevalence in AOUP was lower than that estimated in a multicenter study among HCWs of 105 secondary care health organizations in the UK, between the beginning of September 2020 and the end of April 2021 (44). However, by January 2022, HCWs were exposed to a new variant (i.e., Omicron) that from December 2021 spread aggressively worldwide among the vaccinated healthcare force, rapidly becoming dominant and increasing the risk of re-infections (45).

To date, literature studies investigating the role of significant determinants on SARS-CoV-2 infections in HCWs have conflicting results on the possible role of age (13, 44). Our results show an increased risk of infection for ≤30 year age class HCWs, in line with other studies (42, 46–48), and this is consistent with younger people having more intense social relationships and higher rates of contact. Another possible reason that could explain this result is that younger HCWs might be more likely to be on the frontline and be more likely to be in charge of direct caregiving of patients (42). However, in our hospital, we did not have evidence that younger HCWs were more involved in direct patient care than the other workers. In addition, younger HCWs, despite having received the same training as all HCWs, could still be more at risk due to less work experience, as also suggested in other studies (48).

Our HCWs population did not show any significant differences between sexes. This is consistent with the results reported in other studies (49, 50).

Regarding the job title, a slight but significant increase in the risk of infection was identified for the HCWs (allied health professionals, nurses, physicians, and residents), whose work activity usually involves direct contact with patients, compared to other healthcare and non-healthcare personnel. This result is in agreement with those recently reported in a systematic review and meta-analysis of 54 studies that showed an increased risk of being positive for frontline HCWs (51).

Moving on to the clinical presentation, most HCWs showed mild SARS-CoV-2 infection with few hospitalizations (0.4%), limited to the first two phases of the pre-vaccination era, and no deaths occurred. In particular, during phase 1, 6.7% of positive HCWs were hospitalized, a higher percentage than that reported by other authors (28, 30, 34) but lower than that recorded (8.6%) among the HCWs from the others Regione Veneto health authorities in that period (32). In a meta-analysis of 97 studies that assessed infection among HCWs, 5% of COVID-19 cases in HCWs had severe complications, and 0.5% of HCWs died (5). In phase 2, hospitalizations in AOUP amounted to 0.9%. A study conducted in nine European countries from 31 January 2020 to 13 January 2021 showed an increased adjusted risk of COVID-19 requiring hospitalization or ICU admission in HCWs compared to non-HCWs, respectively, of 1.8 (95% CI 1.2–2.7) and 1.9 (95% CI 1.1–3.2) (52).

Overall, 24.5% of total infections were asymptomatic. Interestingly, our results showed significant differences among study phases with the higher probability of being asymptomatic during phase 1, with a percentage of asymptomatic cases (48%) in line with literature data (53), perhaps, at least partially, justified in the early pandemic stages also by a lower knowledge and awareness of COVID19-related symptoms by both workers and occupational physicians who collected clinical information. A meta-analysis conducted between 01 January 2020 and 02 April 2021 estimated 35.1% of asymptomatic infections in more than 350 studies and 38.5% in 81 studies carried out in healthcare facilities (54). Data from the literature (55) highlighted that centers adopting a screening approach with frequent testing and fast turnaround, such as our center, were more likely to detect a higher number of asymptomatic infections.

Multivariate analysis showed a higher probability of being asymptomatic for men, primary-cycle vaccinated and with a booster dose of vaccine, and those who have already had previous infection. Indeed, vaccinated HCWs are known to have a significantly lower incidence of symptomatic and asymptomatic SARS-CoV-2 infections compared with unvaccinated HCWs (56). In addition, previous SARS-CoV-2 infections are known to reduce the risk of BI (13). As in our study, Methi et al. (57) found that asymptomatic cases had a higher chance of being men. The authors speculated that this result could be an example of men having a higher threshold of reporting symptoms (58).

Fever, upper airway symptoms, myalgia/arthralgia, and headache were the more frequently reported acute phase symptoms. Headache and myalgia/arthralgia were significantly more frequent in women. Indeed, several studies on the long COVID syndrome have identified headache, myalgia (i.e., muscle/body pain), and joint pain as frequently reported symptoms among women (59–61). Unvaccinated HCWs developed more systemic symptoms, e.g., fever, than the vaccinated ones. HCWs vaccinated with the booster dose had a significant reduction in the occurrence of myalgia/arthralgia compared to the two-dose vaccinated. These data are in line with those from the literature that showed vaccine effectiveness against symptomatic infection and severe COVID-19 (62). Overall, during the study period, the symptoms reported by HCWs changed significantly among the five study phases, confirming that the clinical presentation in symptomatic SARS-CoV-2 infections has evolved (20). Indeed, vaccination, immunity from prior infection, and the emergence of the Omicron variant seem to cause a milder clinical presentation (63). However, surveillance of HCWs in AOUP is still going on to evaluate possible post-acute and long-term sequelae of SARS-CoV-2 infection among HCWs (64). Recent studies showed significant long-term persistent symptoms and functional impairment, even in non-hospitalized patients with COVID-19 (65) and occupational settings (66), highlighting the central role of the occupational physician in monitoring workers more closely in the months following primary COVID-19 illness.

### Strengthens and limitations

This study has some strengthens. To the best of our knowledge, this is the first study that analyzed the risk of infection and the clinical presentation of SARS-CoV-2 in HCWs for up to 27 months (i.e., from 15 March 2020 to 06 June 2022), focusing on different pandemic phases related to vaccination and emergence of viral variants. This single-center study involves one of the largest Italian University Hospitals with a large sample of HCWs (health and non-health personnel) routinely and stringently tested for SARS-CoV-2, thus providing reliable estimates of infection rates. In addition, we believe that our data are robust because they emerged from tests always carried out with the rt-PCR method (nasal-pharyngeal or salivary). In fact, when the rapid test was used for the SARS-CoV-2 antigen detection, the confirmation by subsequent rt-PCR test was always performed. This study differentiated between occupational and non-occupational infections by contact tracing, as stated by the Italian occupational compensation scheme. Furthermore, for the contact tracing activity, we did not use a questionnaire intrinsically affected by recall bias, but we performed the activity by direct phone contact with HCWs.

This study has also some limitations. First, since this is a single-center study, it could have limited generalizability issues. Second, those HCWs absent for the entire period and those not yet vaccinated (with at least one dose) at the end of the study period were excluded from the analysis. However, according to the Italian legislation related to the pandemic period, unvaccinated HCWs were suspended from work and remained at home, thus presenting a different risk of infection than that of other health professionals. Third, some HCWs may have intentionally overlooked some source of SARS-CoV-2 infection outside the workplace to access the occupational compensation scheme. However, in the case of coexistent exposures (both in and out of the workplace), our approach was to treat all these infections as occupational. Another limitation was regarding the analysis of the dominant SARS-CoV-2 variant during the study period due to the limited capacity of the DNA sequencing facilities in our center that were mainly dedicated to the analyses of clusters. However, for the considerations presented in this study, the knowledge of the circulating and prevalent variants in a given period/phase was taken into account, as derived from the data regularly communicated at the national and local levels.

## Conclusion

Our analyses provided accurate information on the risk and the determinants of SARS-CoV-2 infection among AOUP HCWs in relation to the different pandemic phases. Our data point out that, besides the availability of protective devices and the implementation of safety protocols, the screening activity on all hospital staff, in particular in the presence of high viral circulation, allowed for the early detection of asymptomatic infected subjects, thus limiting the presence and spreading of clusters inside the hospital wards. However, the control of exposure outside of the workplace also appears to be necessary to limit the nosocomial spread of SARS-CoV-2 among HCWs. The risk of infection was influenced by age, job title, vaccination status, previous infections, and specific pandemic phases that were related to the emergence of new viral variants. During the study period, the clinical presentation in positive HCWs has evolved in relation to vaccination status and the spread of different variants causing less severe disease. Indeed, SARS-CoV-2 vaccination reduced infection spread in working and living environments and the probability of symptomatic COVID-19, demonstrating again its paramount value as a preventive tool for occupational and public health. The results of this study conducted over a long time period across different pandemic phases, characterized by the vaccination campaign and the emerging of new variants, allow us to better identify how the different determinants of infection vary over time. Therefore, based on the aforementioned considerations, hospital administrations will be able to promptly activate proper preventive measures and infection surveillance in future epidemic exacerbations to reduce the spread of COVID-19, especially in vulnerable environments such as hospitals, where HCWs play a critical role in the overall community.

## Data availability statement

The datasets presented in this article are not readily available because the data are not publicly available due to ethical and legal restrictions, as participants of this study did not agree for their data to be shared publicly. Requests to access the datasets should be directed to filippo.liviero@unipd.it.

## Ethics statement

The study was approved by the Italian National Institute of Infectious Diseases (INMI) Lazzaro Spallanzani and the local Research Ethics Committee: Comitato Etico Territoriale Area Centro—Est Veneto (CET-ACEV)—Azienda Ospedale Università di Padova (288n/AO/22). Date were collected during routine health surveillance carried out in compliance with Legislative Decree 81/08 and European Community Directive 90/679. Patient consent was waived since according to Italian privacy law (Legislative Decree 101/2018) patients’ data routinely collected by the Italian National Health Service (NHS) can be used for scientific purposes within the frame of approved studies/protocols, provided sensitive information is anonymized. The studies were conducted in accordance with the local legislation and institutional requirements. Written informed consent for participation was not required from the participants or the participants’ legal guardians/next of kin in accordance with the national legislation and institutional requirements.

## Author contributions

FL, MS, and AM: conceptualization. PF, CC, SC, and VB: methodology. PF: formal analysis. AV, FF, MB, AB, LF, PM, FL, and MS: investigation. AV, FF, MB, AB, LF, PM, FL, and MS: data curation. FF, MB, AB, LF, PF, FL, and MS: writing—original draft preparation. FL, MS, VB, and AM: writing—review and editing. AM: supervision. All authors contributed to the article and approved the submitted version.
