# Supplementary material for: The impact of SARS-CoV-2 on healthcare workers of a large University Hospital in the Veneto Region: risk of infection and clinical presentation in relation to different pandemic phases and some relevant determinants
Source: Front Public Health. 2023 Nov 30;11:1250911. doi: 10.3389/fpubh.2023.1250911 (PMC10720910; doi:10.3389/fpubh.2023.1250911)
Supplement: Supplementary file 1 [file Data_Sheet_1.docx]

**Supplementary Materials**

**Figure S1.** Schematic representation of transmission cluster amongst HCWs (and their relatives) stratified according to the source, age, professional role, ward and symptoms.


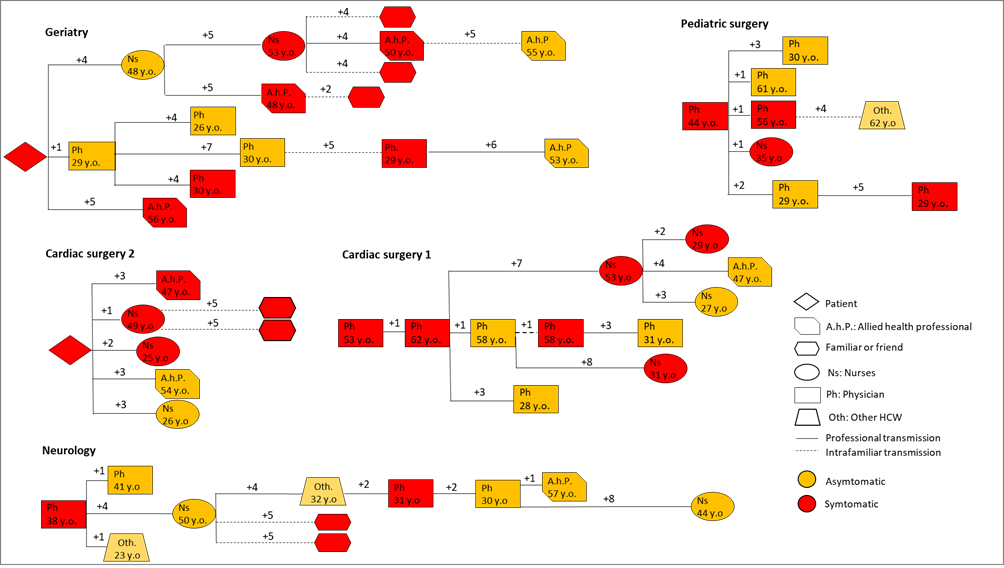


Every symbol refers to a single case SARS-CoV-2 tested positive involved in one of the five clusters (Pediatric surgery; Neurology; Cardiac surgery 1 and 2; Geriatric). Red color indicates symptomatic cases; yellow color indicates asymptomatic cases. Inside the symbol (for HCWs), it is reported age and professional role (Ns: Nurse; A.h.p: Allied health professional; Ph: Physician; R.d.: Resident doctors; Oth hcw: others HCWs). The lines represent the transmission contact (solid line: professional transmission; dashed line: intrafamily transmission). The numbers reported above the transmission lines represent the time elapsed from the exposure to the positive nasopharyngeal swab of HCW or family member.

**Table S1.** Multivariate logistic regression analysis investigating the risk of infection by contact with a positive colleague, by contact with a positive patient and out of workplace stratified by some relevant characteristics.

|  | **Positivity to SARS-CoV-2** | **Infection by contact with a positive colleague (n.324)** | | | **Infection out of workplace (n.1,417)** | | | **Infection by patient (n.196)** | | |
| --- | --- | --- | --- | --- | --- | --- | --- | --- | --- | --- |
|  | **(n.4,005)** | **N** | **(%)** | **adjOR** | **N** | **(%)** | **adjOR** | **N** | **(%)** | **adjOR** |
| **Gender** |  |  |  |  |  |  |  |  |  |  |
| *Male* | 1,236 | 118 | (9.5) | 1.15 (0.88-1.50) | 420 | (34.0) | 0.99 (0.85-1.16) | 53 | (4.3) | 0.94 (0.66-1.33) |
| *Female* | 2,769 | 206 | (7.4) | (ref) | 997 | (36.0) | (ref) | 143 | (5.2) | (ref) |
| **Age groups** |  |  |  |  |  |  |  |  |  |  |
| *<=30* | 1,203 | 129 | (10.7) | 1.44 (1.00-2.07) | 318 | (26.4) | (ref) | 64 | (5.3) | **1.73 (1.09-2.77)** |
| *31-49* | 1,527 | 93 | (6.1) | (ref) | 615 | (40.3) | **1.73 (1.42-2.12)** | 74 | (4.8) | 1.15 (0.79-1.67) |
| *50+* | 1,275 | 102 | (8.0) | 1.30 (0.95-1.79) | 484 | (38.0) | **1.53 (1.22-1.91)** | 58 | (4.5) | (ref) |
| **Job title** |  |  |  |  |  |  |  |  |  |  |
| *Allied health professionals* | 548 | 37 | (6.8) | (ref) | 170 | (31.0) | (ref) | 48 | (8.8) | **1.85 (1.05-3.25)** |
| *Nurses* | 1,460 | 97 | (6.6) | 1.04 (0.68-1.60) | 569 | (39.0) | **1.53 (1.23-1.91)** | 85 | (5.8) | 1.23 (0.73-2.07) |
| *Phisicians* | 514 | 38 | (7.4) | 1.18 (0.70-1.98) | 194 | (37.7) | 1.30 (0.99-1.71) | 21 | (4.1) | (ref) |
| *Residents* | 912 | 107 | (11.7) | **1.78 (1.06-2.99)** | 243 | (26.6) | 1.13 (0.84-1.50) | 37 | (4.1) | 0.74 (0.39-1.42) |
| *Other heathcare personnel* | 189 | 8 | (4.2) | 0.76 (0.33-1.75) | 71 | (37.6) | 1.31 (0.91-1.87) | 5 | (2.6) | 0.73 (0.27-2.03) |
| *Other non-healthcare personnel* | 382 | 37 | (9.7) | **2.00 (1.20-3.33)** | 170 | (44.5) | **1.55 (1.17-2.06)** | 0 | (0.0) |  |
| **Cluster** |  |  |  |  |  |  |  |  |  |  |
| *Yes* | 574 | 140 | (24.4) | **5.73 (4.33-7.56)** | 54 | (9.4) | **0.16 (0.12-0.21)** | 86 | (15.0) | **3.27 (2.35-4.55)** |
| *NO* | 3431 | 184 | (5.4) | (ref) | 1363 | (39.7) | (ref) | 110 | (3.2) | (ref) |
| **COVID area** |  |  |  |  |  |  |  |  |  |  |
| *Yes* | 589 | 31 | (5.3) | (ref) | 182 | (30.9) | (ref) | 36 | (6.1) | (ref) |
| *NO* | 3,416 | 293 | (8.6) | **2.59 (1.72-3.91)** | 1,235 | (36.2) | 1.15 (0.94-1.41) | 160 | (4.7) | 1.15 (0.78-1.69) |
| **N. of doses to positivity** |  |  |  |  |  |  |  |  |  |  |
| *0* | 830 | 123 | (14.8) | (ref) | 249 | (30.0) | (ref) | 102 | (12.3) | (ref) |
| *1* | 53 | 2 | (3.8) | 0.88 (0.14-5.50) | 16 | (30.2) | 0.64 (0.28-1.42) | 2 | (3.8) | 0.37 (0.06-2.20) |
| *2* | 344 | 18 | (5.2) | 1.27 (0.30-5.31) | 160 | (46.5) | 1.37 (0.72-2.60) | 12 | (3.5) | 0.38 (0.10-1.46) |
| *3* | 2,778 | 181 | (6.5) | 1.75 (0.39-7.78) | 992 | (35.7) | 1.04 (0.53-2.06) | 80 | (2.9) | 0.39 (0.09-1.68) |
| **Previous infections** |  |  |  |  |  |  |  |  |  |  |
| none | 3,760 | 314 | (8.4) | (ref) | 1,327 | (35.3) | (ref) | 183 | (4.9) | (ref) |
| *<=12months* | 86 | 3 | (3.5) | 0.58 (0.18-1.88) | 29 | (33.7) | 0.88 (0.55-1.41) | 3 | (3.5) | 0.84 (0.25-2.80) |
| 12+*months* | 159 | 7 | (4.4) | 0.81 (0.37-1.80) | 61 | (38.4) | 1.07 (0.76-1.51) | 10 | (6.3) | **2.20 (1.09-4.44)** |
| **Pandemic study phase** |  |  |  |  |  |  |  |  |  |  |
| *Phase 1* | 135 | 49 | (36.3) | **6.65 (1.74-25.47)** | 21 | (15.6) | **0.36 (0.17-0.75)** | 15 | (11.1) | 0.68 (0.21-2.19) |
| *Phase 2* | 652 | 72 | (11.0) | 1.26 (0.34-4.68) | 208 | (31.9) | 0.92 (0.51-1.64) | 83 | (12.7) | 0.86 (0.30-2.44) |
| *Phase 3* | 161 | 8 | (5.0) | (ref) | 78 | (48.4) | (ref) | 8 | (5.0) | (ref) |
| *Phase 4* | 1,505 | 107 | (7.1) | 0.75 (0.29-1.97) | 602 | (40.0) | 0.88 (0.57-1.37) | 52 | (3.5) | 0.67 (0.22-2.12) |
| *Phase 5* | 1,552 | 88 | (5.7) | 0.58 (0.21-1.58) | 508 | (32.7) | **0.61 (0.39-0.97)** | 38 | (2.4) | 0.52 (0.16-1.74) |

Legend: bold indicates statistically significant results.

**Table S2**. Distribution of symptomatic SARS-CoV-2 infections (in absolute number and percentage) by the different study phases and type of symptoms.

| **Symptoms** | **Phase1 (n.70)** | | **Phase2 (n.476)** | | **Phase3 (n.120)** | | **Phase4 (n.1,050)** | | **Phase5 (n.1,308)** | | **Total (n.3,024)** | |
| --- | --- | --- | --- | --- | --- | --- | --- | --- | --- | --- | --- | --- |
|  | **n. %** | | **n. %** | | **n. %** | | **n. %** | | **n. %** | | **n. %** | |
| **Fever** | 52 | 74.3 | 255 | 53.6 | 54 | 45.0 | 297 | 28.3 | 467 | 35.7 | 1,125 | 37.2 |
| **Sore throat** | 9 | 12.9 | 79 | 16.6 | 20 | 16.7 | 424 | 40.4 | 600 | 45.9 | 1,132 | 37.4 |
| **Cough** | 28 | 40.0 | 151 | 31.7 | 34 | 28.3 | 338 | 32.2 | 469 | 35.9 | 1,020 | 33.7 |
| **Rhinorrhea** | 9 | 12.9 | 94 | 19.7 | 55 | 45.8 | 375 | 35.7 | 487 | 37.2 | 1,020 | 33.7 |
| Headache | 14 | 20.0 | 123 | 25.8 | 37 | 30.8 | 199 | 19.0 | 203 | 15.5 | 576 | 19.0 |
| Myalgia/arthralgia | 9 | 12.9 | 161 | 33.8 | 23 | 19.2 | 127 | 12.1 | 163 | 12.5 | 483 | 16.0 |
| Nasal obstruction | 1 | 1.4 | 64 | 13.4 | 18 | 15.0 | 138 | 13.1 | 224 | 17.1 | 445 | 14.7 |
| Asthenia | 14 | 20.0 | 92 | 19.3 | 17 | 14.2 | 113 | 10.8 | 159 | 12.2 | 395 | 13.1 |
| Ageusia/anosmia | 17 | 24.3 | 94 | 19.7 | 12 | 10.0 | 22 | 2.1 | 7 | 0.5 | 152 | 5.0 |
| Diarrhea | 1 | 1.4 | 25 | 5.3 | 0 | 0.0 | 11 | 1.0 | 12 | 0.9 | 49 | 1.6 |
| Dyspnea | 11 | 15.7 | 13 | 2.7 | 3 | 2.5 | 5 | 0.5 | 4 | 0.3 | 36 | 1.2 |
| Nausea/vomit |  |  | 21 | 4.4 | 1 | 0.8 | 6 | 0.6 | 14 | 1.1 | 42 | 1.4 |
| Chest pain |  |  | 3 | 0.6 |  |  | 5 | 0.5 | 1 | 0.1 | 9 | 0.3 |
| Anorexia |  |  | 3 | 0.6 |  |  | 1 | 0.1 |  |  | 4 | 0.1 |
| Confusion |  |  | 4 | 0.8 |  |  | 1 | 0.1 | 1 | 0.1 | 6 | 0.2 |
| Rush |  |  | 4 | 0.8 |  |  |  |  |  |  | 4 | 0.1 |
| Tachycardia |  |  | 2 | 0.4 |  |  | 2 | 0.2 | 1 | 0.1 | 5 | 0.2 |

Legend: bold indicates the most frequent symptoms reported by HCWs during the acute phase of infection.
